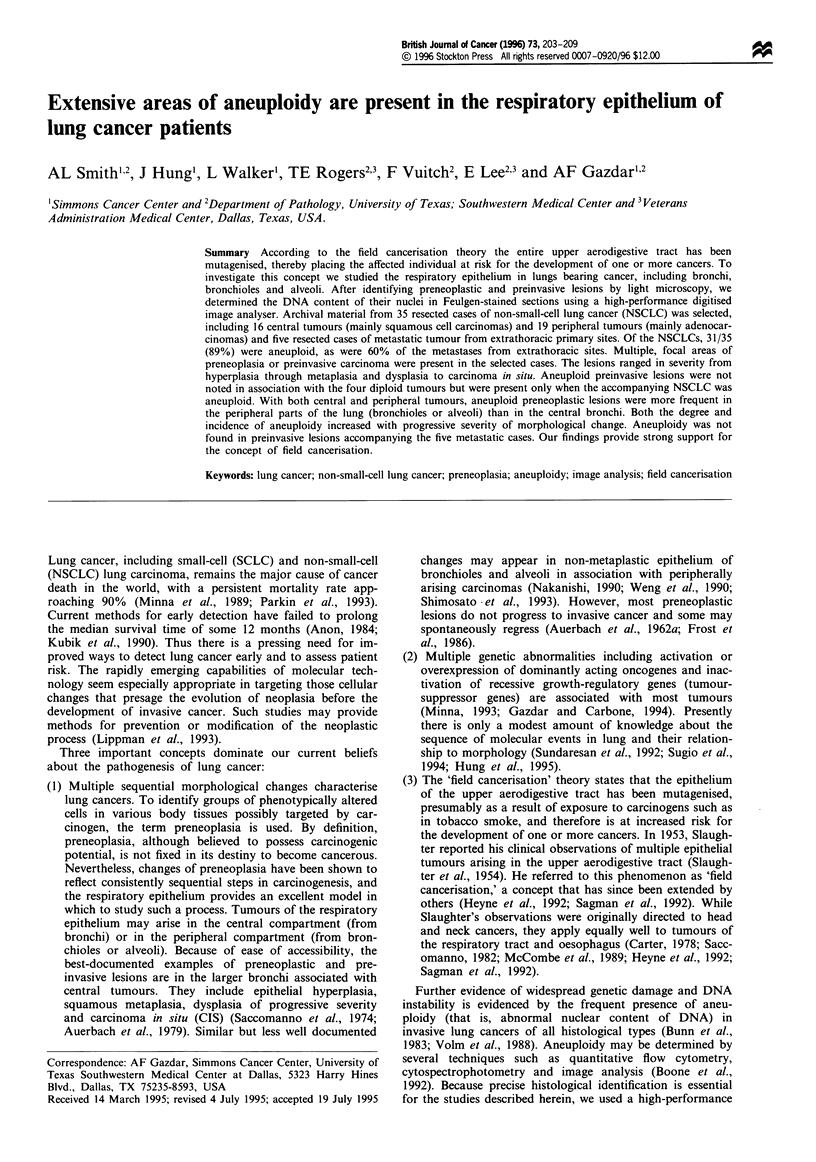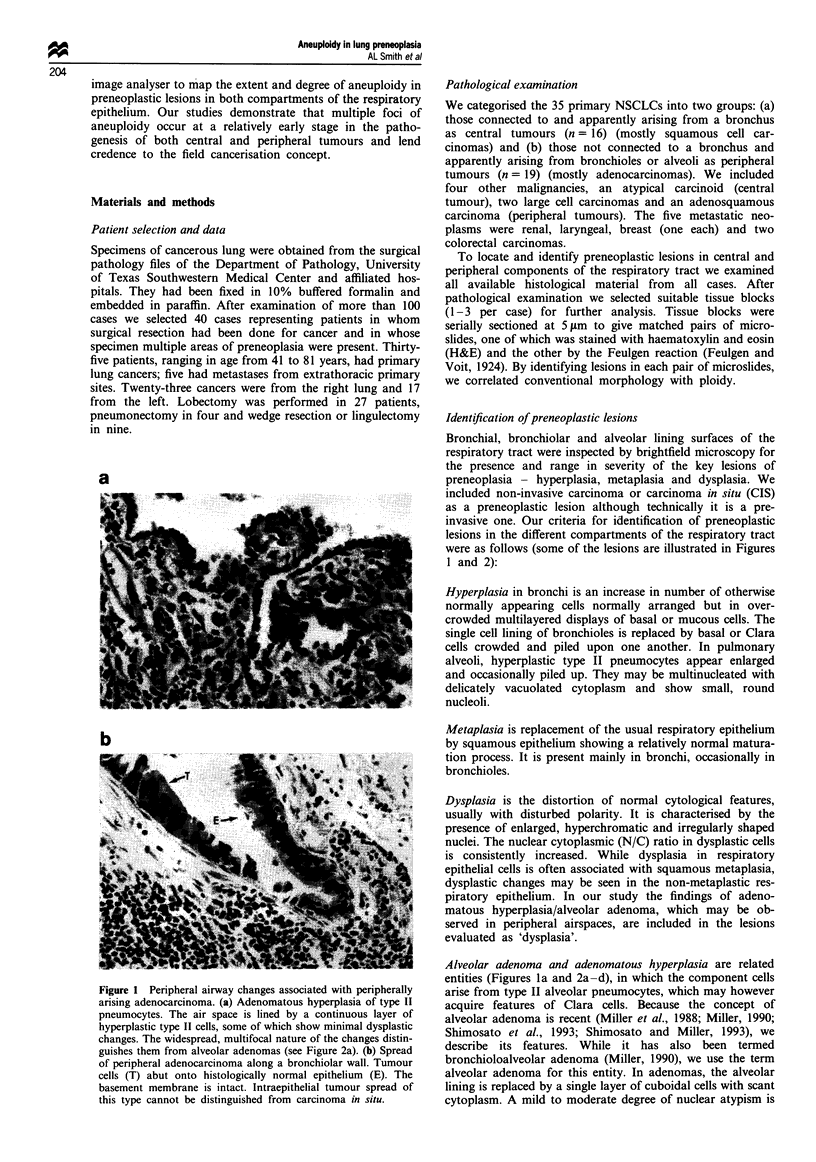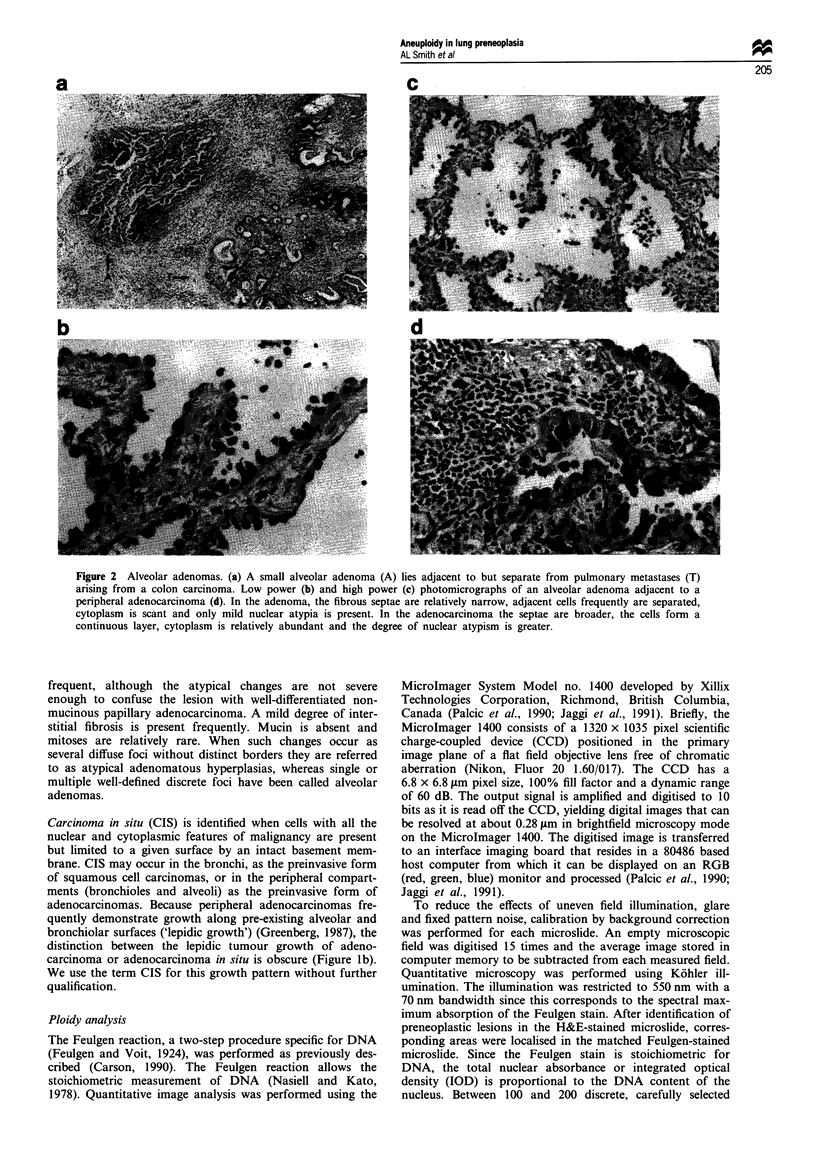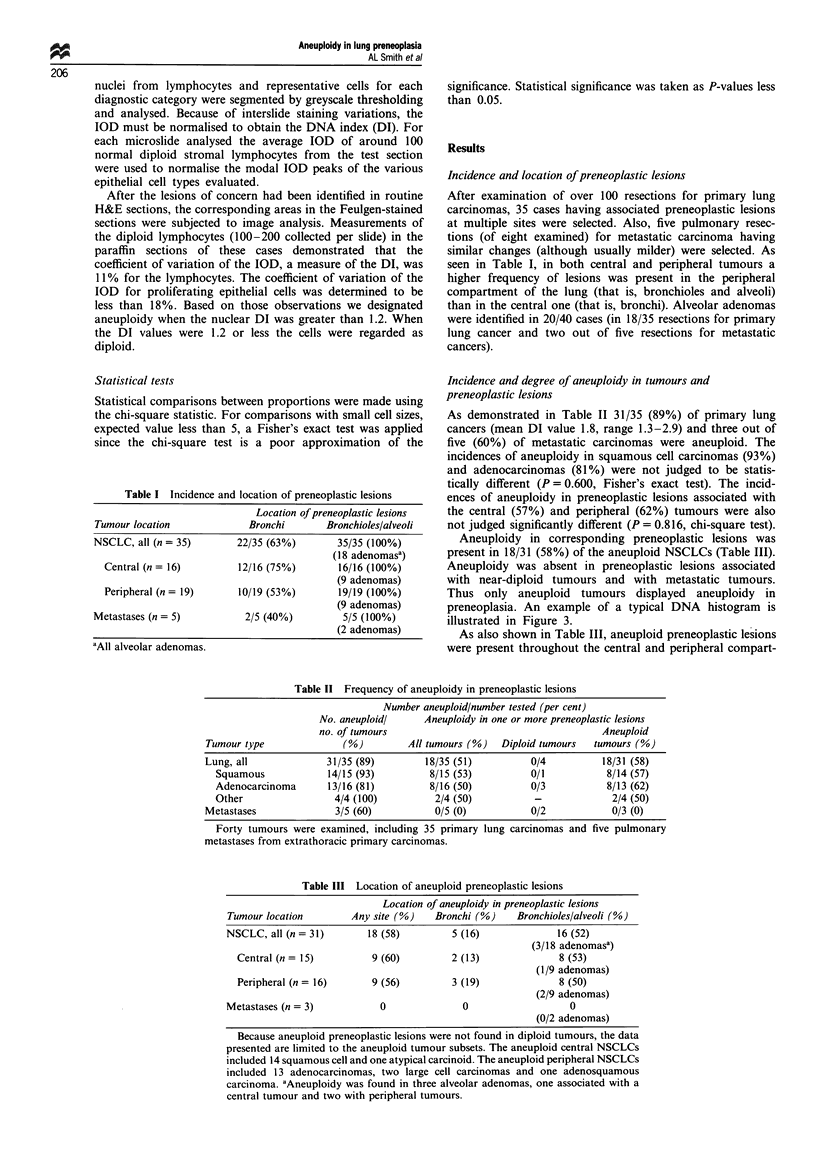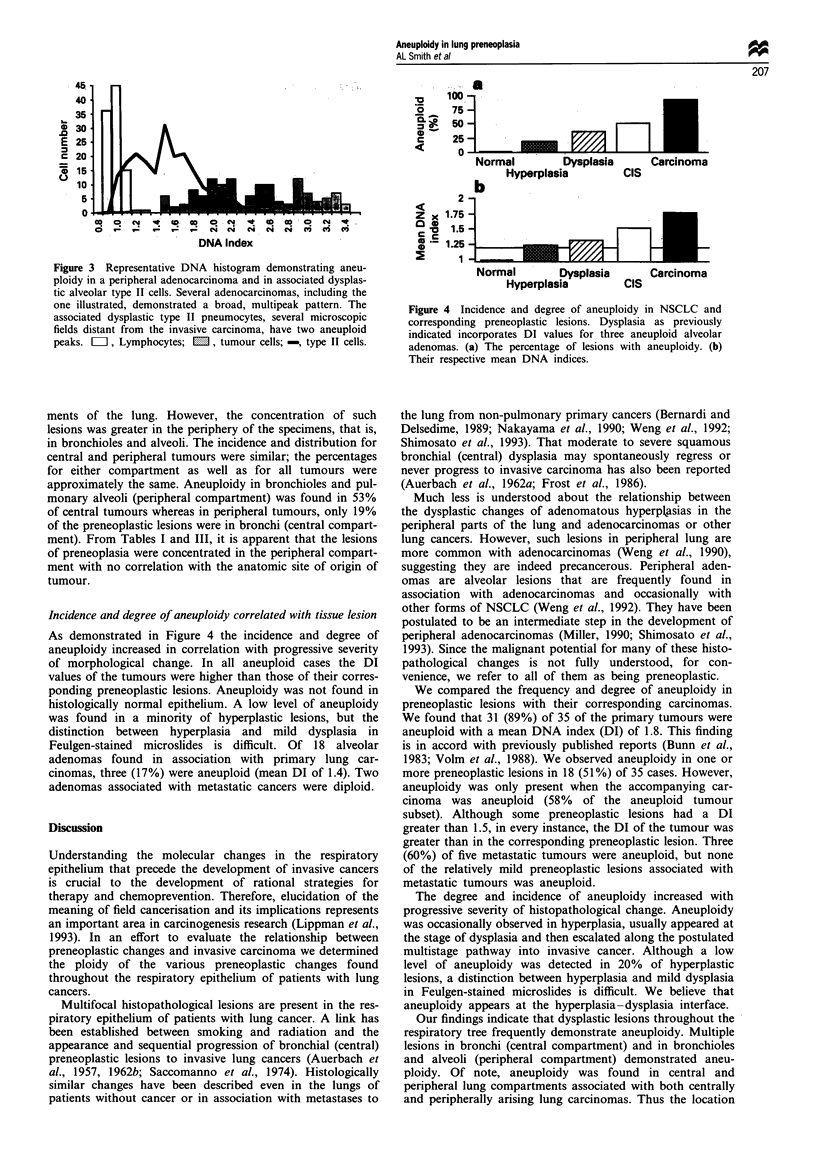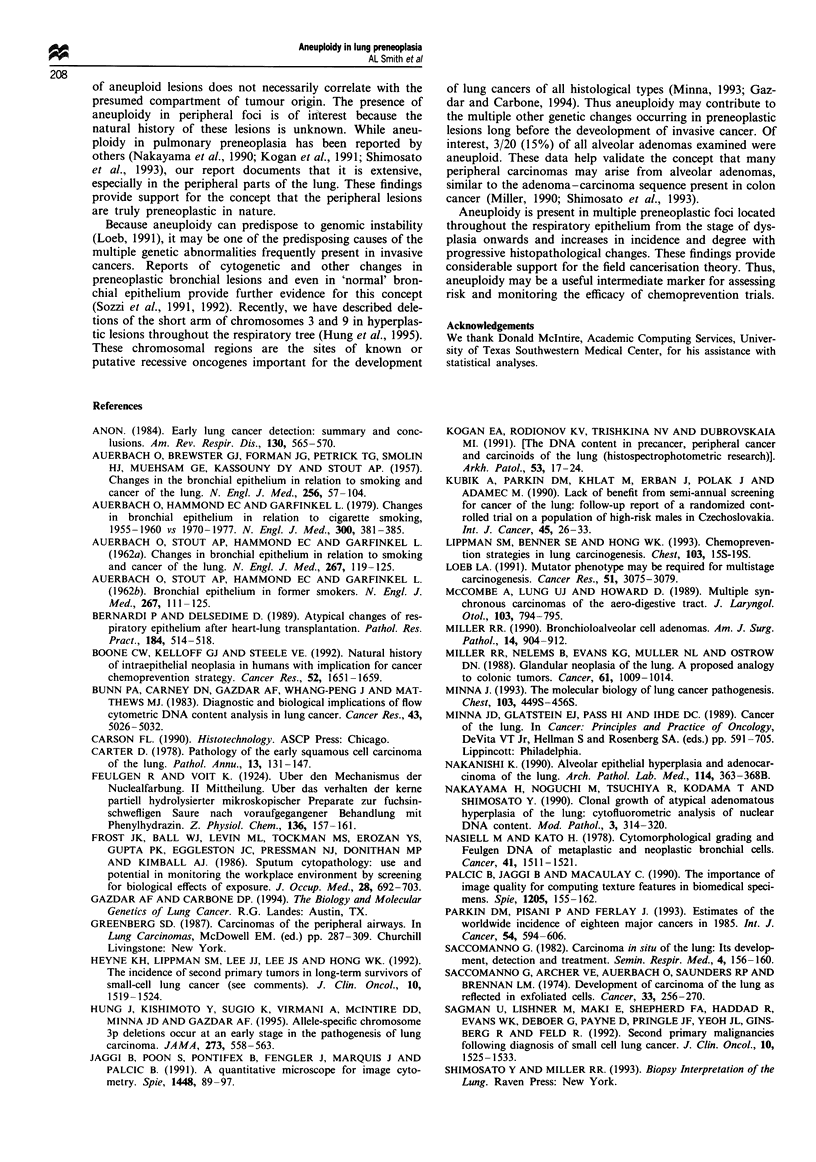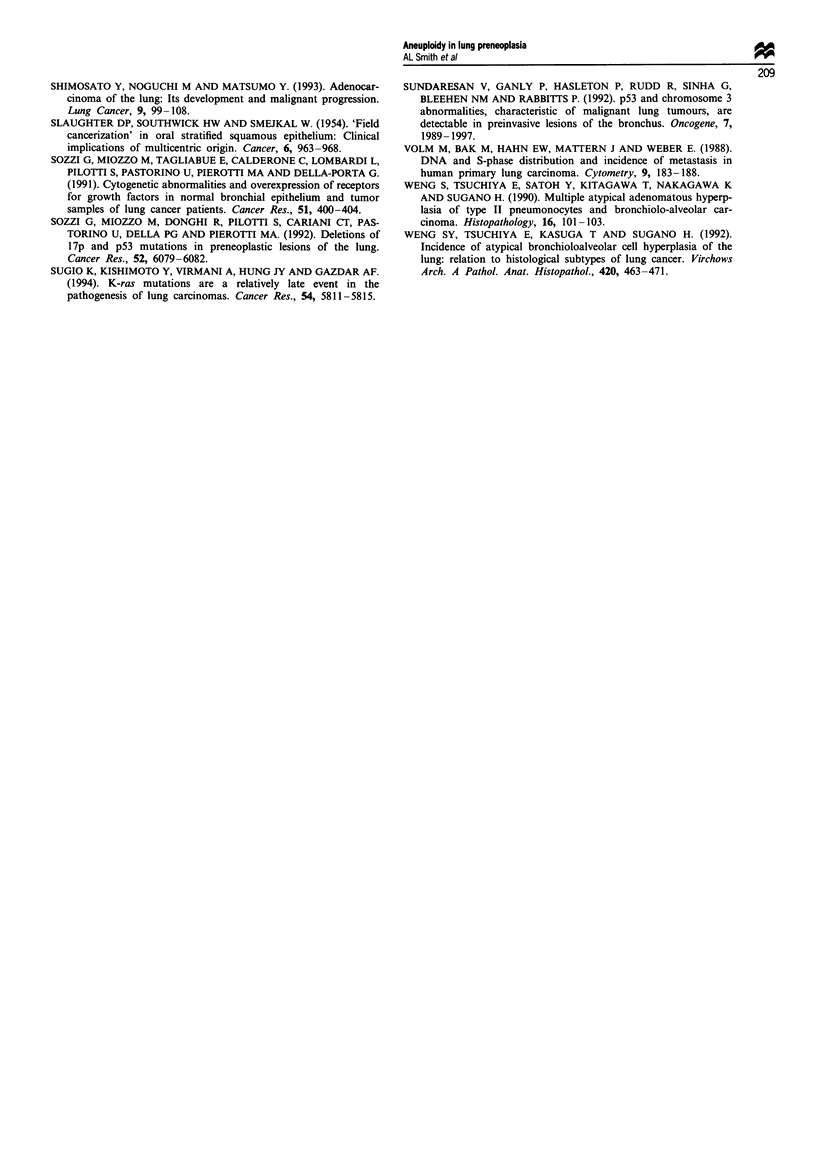# Extensive areas of aneuploidy are present in the respiratory epithelium of lung cancer patients.

**DOI:** 10.1038/bjc.1996.36

**Published:** 1996-01

**Authors:** A. L. Smith, J. Hung, L. Walker, T. E. Rogers, F. Vuitch, E. Lee, A. F. Gazdar

**Affiliations:** Simmons Cancer Center, University of Texas, Dallas, USA.

## Abstract

**Images:**


British Journal of Cancer (1996) 73, 203–209
© 1996 Stockton Press All rights reserved 0007–0920/96 $12.00

### Extensive areas of aneuploidy are present in the respiratory epithelium of lung cancer patients

AL Smith[1,2], J Hung[1], L Walker[1], TE Rogers[2,3], F Vuitch[2], E Lee[2,3] and AF Gazdar[1,2]

[1]*Simmons Cancer Center and* [2]*Department of Pathology, University of Texas; Southwestern Medical Center and* [3]*Veterans Administration Medical Center, Dallas, Texas, USA.*

**Summary** According to the field cancerisation theory the entire upper aerodigestive tract has been mutagenised, thereby placing the affected individual at risk for the development of one or more cancers. To investigate this concept we studied the respiratory epithelium in lungs bearing cancer, including bronchi, bronchioles and alveoli. After identifying preneoplastic and preinvasive lesions by light microscopy, we determined the DNA content of their nuclei in Feulgen-stained sections using a high-performance digitised image analyser. Archival material from 35 resected cases of non-small-cell lung cancer (NSCLC) was selected, including 16 central tumours (mainly squamous cell carcinomas) and 19 peripheral tumours (mainly adenocarcinomas) and five resected cases of metastatic tumour from extrathoracic primary sites. Of the NSCLCs, 31/35 (89%) were aneuploid, as were 60% of the metastases from extrathoracic sites. Multiple, focal areas of preneoplasia or preinvasive carcinoma were present in the selected cases. The lesions ranged in severity from hyperplasia through metaplasia and dysplasia to carcinoma *in situ*. Aneuploid preinvasive lesions were not noted in association with the four diploid tumours but were present only when the accompanying NSCLC was aneuploid. With both central and peripheral tumours, aneuploid preneoplastic lesions were more frequent in the peripheral parts of the lung (bronchioles or alveoli) than in the central bronchi. Both the degree and incidence of aneuploidy increased with progressive severity of morphological change. Aneuploidy was not found in preinvasive lesions accompanying the five metastatic cases. Our findings provide strong support for the concept of field cancerisation.

**Keywords:** lung cancer; non-small-cell lung cancer; preneoplasia; aneuploidy; image analysis; field cancerisation

Lung cancer, including small-cell (SCLC) and non-small-cell (NSCLC) lung carcinoma, remains the major cause of cancer death in the world, with a persistent mortality rate approaching 90% (Minna *et al.*, 1989; Parkin *et al.*, 1993). Current methods for early detection have failed to prolong the median survival time of some 12 months (Anon, 1984; Kubik *et al.*, 1990). Thus there is a pressing need for improved ways to detect lung cancer early and to assess patient risk. The rapidly emerging capabilities of molecular technology seem especially appropriate in targeting those cellular changes that presage the evolution of neoplasia before the development of invasive cancer. Such studies may provide methods for prevention or modification of the neoplastic process (Lippman *et al.*, 1993).

Three important concepts dominate our current beliefs about the pathogenesis of lung cancer:

(1) Multiple sequential morphological changes characterise lung cancers. To identify groups of phenotypically altered cells in various body tissues possibly targeted by carcinogen, the term preneoplasia is used. By definition, preneoplasia, although believed to possess carcinogenic potential, is not fixed in its destiny to become cancerous. Nevertheless, changes of preneoplasia have been shown to reflect consistently sequential steps in carcinogenesis, and the respiratory epithelium provides an excellent model in which to study such a process. Tumours of the respiratory epithelium may arise in the central compartment (from bronchi) or in the peripheral compartment (from bronchioles or alveoli). Because of ease of accessibility, the best-documented examples of preneoplastic and preinvasive lesions are in the larger bronchi associated with central tumours. They include epithelial hyperplasia, squamous metaplasia, dysplasia of progressive severity and carcinoma *in situ* (CIS) (Saccomanno *et al.*, 1974; Auerbach *et al.*, 1979). Similar but less well documented changes may appear in non-metaplastic epithelium of bronchioles and alveoli in association with peripherally arising carcinomas (Nakanishi, 1990; Weng *et al.*, 1990; Shimosato *et al.*, 1993). However, most preneoplastic lesions do not progress to invasive cancer and some may spontaneously regress (Auerbach *et al.*, 1962*a*; Frost *et al.*, 1986).

(2) Multiple genetic abnormalities including activation or overexpression of dominantly acting oncogenes and inactivation of recessive growth-regulatory genes (tumour-suppressor genes) are associated with most tumours (Minna, 1993; Gazdar and Carbone, 1994). Presently there is only a modest amount of knowledge about the sequence of molecular events in lung and their relationship to morphology (Sundaresan *et al.*, 1992; Sugio *et al.*, 1994; Hung *et al.*, 1995).

(3) The 'field cancerisation' theory states that the epithelium of the upper aerodigestive tract has been mutagenised, presumably as a result of exposure to carcinogens such as in tobacco smoke, and therefore is at increased risk for the development of one or more cancers. In 1953, Slaughter reported his clinical observations of multiple epithelial tumours arising in the upper aerodigestive tract (Slaughter *et al.*, 1954). He referred to this phenomenon as 'field cancerisation,' a concept that has since been extended by others (Heyne *et al.*, 1992; Sagman *et al.*, 1992). While Slaughter's observations were originally directed to head and neck cancers, they apply equally well to tumours of the respiratory tract and oesophagus (Carter, 1978; Saccomanno, 1982; McCombe *et al.*, 1989; Heyne *et al.*, 1992; Sagman *et al.*, 1992).

Further evidence of widespread genetic damage and DNA instability is evidenced by the frequent presence of aneuploidy (that is, abnormal nuclear content of DNA) in invasive lung cancers of all histological types (Bunn *et al.*, 1983; Volm *et al.*, 1988). Aneuploidy may be determined by several techniques such as quantitative flow cytometry, cytospectrophotometry and image analysis (Boone *et al.*, 1992). Because precise histological identification is essential for the studies described herein, we used a high-performance

Correspondence: AF Gazdar, Simmons Cancer Center, University of Texas Southwestern Medical Center at Dallas, 5323 Harry Hines Blvd., Dallas, TX 75235-8593, USA
Received 14 March 1995; revised 4 July 1995; accepted 19 July 1995

image analyser to map the extent and degree of aneuploidy in preneoplastic lesions in both compartments of the respiratory epithelium. Our studies demonstrate that multiple foci of aneuploidy occur at a relatively early stage in the pathogenesis of both central and peripheral tumours and lend credence to the field cancerisation concept.

### Materials and methods

### *Patient selection and data*

Specimens of cancerous lung were obtained from the surgical pathology files of the Department of Pathology, University of Texas Southwestern Medical Center and affiliated hospitals. They had been fixed in 10% buffered formalin and embedded in paraffin. After examination of more than 100 cases we selected 40 cases representing patients in whom surgical resection had been done for cancer and in whose specimen multiple areas of preneoplasia were present. Thirty-five patients, ranging in age from 41 to 81 years, had primary lung cancers; five had metastases from extrathoracic primary sites. Twenty-three cancers were from the right lung and 17 from the left. Lobectomy was performed in 27 patients, pneumonectomy in four and wedge resection or lingulectomy in nine.

**Figure 1** Peripheral airway changes associated with peripherally arising adenocarcinoma. (**a**) Adenomatous hyperplasia of type II pneumocytes. The air space is lined by a continuous layer of hyperplastic type II cells, some of which show minimal dysplastic changes. The widespread, multifocal nature of the changes distinguishes them from alveolar adenomas (see Figure 2a). (**b**) Spread of peripheral adenocarcinoma along a bronchiolar wall. Tumour cells (T) abut onto histologically normal epithelium (E). The basement membrane is intact. Intraepithelial tumour spread of this type cannot be distinguished from carcinoma *in situ*.

### *Pathological examination*

We categorised the 35 primary NSCLCs into two groups: (a) those connected to and apparently arising from a bronchus as central tumours ($n = 16$) (mostly squamous cell carcinomas) and (b) those not connected to a bronchus and apparently arising from bronchioles or alveoli as peripheral tumours ($n = 19$) (mostly adenocarcinomas). We included four other malignancies, an atypical carcinoid (central tumour), two large cell carcinomas and an adenosquamous carcinoma (peripheral tumours). The five metastatic neoplasms were renal, laryngeal, breast (one each) and two colorectal carcinomas.

To locate and identify preneoplastic lesions in central and peripheral components of the respiratory tract we examined all available histological material from all cases. After pathological examination we selected suitable tissue blocks (1–3 per case) for further analysis. Tissue blocks were serially sectioned at 5 $\mu$m to give matched pairs of microslides, one of which was stained with haematoxylin and eosin (H&E) and the other by the Feulgen reaction (Feulgen and Voit, 1924). By identifying lesions in each pair of microslides, we correlated conventional morphology with ploidy.

### *Identification of preneoplastic lesions*

Bronchial, bronchiolar and alveolar lining surfaces of the respiratory tract were inspected by brightfield microscopy for the presence and range in severity of the key lesions of preneoplasia – hyperplasia, metaplasia and dysplasia. We included non-invasive carcinoma or carcinoma *in situ* (CIS) as a preneoplastic lesion although technically it is a pre-invasive one. Our criteria for identification of preneoplastic lesions in the different compartments of the respiratory tract were as follows (some of the lesions are illustrated in Figures 1 and 2):

*Hyperplasia* in bronchi is an increase in number of otherwise normally appearing cells normally arranged but in overcrowded multilayered displays of basal or mucous cells. The single cell lining of bronchioles is replaced by basal or Clara cells crowded and piled upon one another. In pulmonary alveoli, hyperplastic type II pneumocytes appear enlarged and occasionally piled up. They may be multinucleated with delicately vacuolated cytoplasm and show small, round nucleoli.

*Metaplasia* is replacement of the usual respiratory epithelium by squamous epithelium showing a relatively normal maturation process. It is present mainly in bronchi, occasionally in bronchioles.

*Dysplasia* is the distortion of normal cytological features, usually with disturbed polarity. It is characterised by the presence of enlarged, hyperchromatic and irregularly shaped nuclei. The nuclear cytoplasmic (N/C) ratio in dysplastic cells is consistently increased. While dysplasia in respiratory epithelial cells is often associated with squamous metaplasia, dysplastic changes may be seen in the non-metaplastic respiratory epithelium. In our study the findings of adenomatous hyperplasia/alveolar adenoma, which may be observed in peripheral airspaces, are included in the lesions evaluated as 'dysplasia'.

*Alveolar adenoma and adenomatous hyperplasia* are related entities (Figures 1a and 2a–d), in which the component cells arise from type II alveolar pneumocytes, which may however acquire features of Clara cells. Because the concept of alveolar adenoma is recent (Miller *et al*., 1988; Miller, 1990; Shimosato *et al*., 1993; Shimosato and Miller, 1993), we describe its features. While it has also been termed bronchioloalveolar adenoma (Miller, 1990), we use the term alveolar adenoma for this entity. In adenomas, the alveolar lining is replaced by a single layer of cuboidal cells with scant cytoplasm. A mild to moderate degree of nuclear atypism is

**Figure 2** Alveolar adenomas. (**a**) A small alveolar adenoma (A) lies adjacent to but separate from pulmonary metastases (T) arising from a colon carcinoma. Low power (**b**) and high power (**c**) photomicrographs of an alveolar adenoma adjacent to a peripheral adenocarcinoma (**d**). In the adenoma, the fibrous septae are relatively narrow, adjacent cells frequently are separated, cytoplasm is scant and only mild nuclear atypia is present. In the adenocarcinoma the septae are broader, the cells form a continuous layer, cytoplasm is relatively abundant and the degree of nuclear atypism is greater.

frequent, although the atypical changes are not severe enough to confuse the lesion with well-differentiated non-mucinous papillary adenocarcinoma. A mild degree of interstitial fibrosis is present frequently. Mucin is absent and mitoses are relatively rare. When such changes occur as several diffuse foci without distinct borders they are referred to as atypical adenomatous hyperplasias, whereas single or multiple well-defined discrete foci have been called alveolar adenomas.

*Carcinoma in situ* (CIS) is identified when cells with all the nuclear and cytoplasmic features of malignancy are present but limited to a given surface by an intact basement membrane. CIS may occur in the bronchi, as the preinvasive form of squamous cell carcinomas, or in the peripheral compartments (bronchioles and alveoli) as the preinvasive form of adenocarcinomas. Because peripheral adenocarcinomas frequently demonstrate growth along pre-existing alveolar and bronchiolar surfaces ('lepidic growth') (Greenberg, 1987), the distinction between the lepidic tumour growth of adenocarcinoma or adenocarcinoma *in situ* is obscure (Figure 1b). We use the term CIS for this growth pattern without further qualification.

### *Ploidy analysis*

The Feulgen reaction, a two-step procedure specific for DNA (Feulgen and Voit, 1924), was performed as previously described (Carson, 1990). The Feulgen reaction allows the stoichiometric measurement of DNA (Nasiell and Kato, 1978). Quantitative image analysis was performed using the MicroImager System Model no. 1400 developed by Xillix Technologies Corporation, Richmond, British Columbia, Canada (Palcic *et al.*, 1990; Jaggi *et al.*, 1991). Briefly, the MicroImager 1400 consists of a 1320 × 1035 pixel scientific charge-coupled device (CCD) positioned in the primary image plane of a flat field objective lens free of chromatic aberration (Nikon, Fluor 20 1.60/017). The CCD has a 6.8 × 6.8 μm pixel size, 100% fill factor and a dynamic range of 60 dB. The output signal is amplified and digitised to 10 bits as it is read off the CCD, yielding digital images that can be resolved at about 0.28 μm in brightfield microscopy mode on the MicroImager 1400. The digitised image is transferred to an interface imaging board that resides in a 80486 based host computer from which it can be displayed on an RGB (red, green, blue) monitor and processed (Palcic *et al.*, 1990; Jaggi *et al.*, 1991).

To reduce the effects of uneven field illumination, glare and fixed pattern noise, calibration by background correction was performed for each microslide. An empty microscopic field was digitised 15 times and the average image stored in computer memory to be subtracted from each measured field. Quantitative microscopy was performed using Köhler illumination. The illumination was restricted to 550 nm with a 70 nm bandwidth since this corresponds to the spectral maximum absorption of the Feulgen stain. After identification of preneoplastic lesions in the H&E-stained microslide, corresponding areas were localised in the matched Feulgen-stained microslide. Since the Feulgen stain is stoichiometric for DNA, the total nuclear absorbance or integrated optical density (IOD) is proportional to the DNA content of the nucleus. Between 100 and 200 discrete, carefully selected nuclei from lymphocytes and representative cells for each diagnostic category were segmented by greyscale thresholding and analysed. Because of interslide staining variations, the IOD must be normalised to obtain the DNA index (DI). For each microslide analysed the average IOD of around 100 normal diploid stromal lymphocytes from the test section were used to normalise the modal IOD peaks of the various epithelial cell types evaluated.

After the lesions of concern had been identified in routine H&E sections, the corresponding areas in the Feulgen-stained sections were subjected to image analysis. Measurements of the diploid lymphocytes (100–200 collected per slide) in the paraffin sections of these cases demonstrated that the coefficient of variation of the IOD, a measure of the DI, was 11% for the lymphocytes. The coefficient of variation of the IOD for proliferating epithelial cells was determined to be less than 18%. Based on those observations we designated aneuploidy when the nuclear DI was greater than 1.2. When the DI values were 1.2 or less the cells were regarded as diploid.

*Statistical tests*

Statistical comparisons between proportions were made using the chi-square statistic. For comparisons with small cell sizes, expected value less than 5, a Fisher's exact test was applied since the chi-square test is a poor approximation of the significance. Statistical significance was taken as $P$-values less than 0.05.

**Table I** Incidence and location of preneoplastic lesions

| Tumour location | *Location of preneoplastic lesions* Bronchi | Bronchioles/alveoli |
|---|---|---|
| NSCLC, all ($n = 35$) | 22/35 (63%) | 35/35 (100%) (18 adenomas[a]) |
| Central ($n = 16$) | 12/16 (75%) | 16/16 (100%) (9 adenomas) |
| Peripheral ($n = 19$) | 10/19 (53%) | 19/19 (100%) (9 adenomas) |
| Metastases ($n = 5$) | 2/5 (40%) | 5/5 (100%) (2 adenomas) |

[a]All alveolar adenomas.

### Results

*Incidence and location of preneoplastic lesions*

After examination of over 100 resections for primary lung carcinomas, 35 cases having associated preneoplastic lesions at multiple sites were selected. Also, five pulmonary resections (of eight examined) for metastatic carcinoma having similar changes (although usually milder) were selected. As seen in Table I, in both central and peripheral tumours a higher frequency of lesions was present in the peripheral compartment of the lung (that is, bronchioles and alveoli) than in the central one (that is, bronchi). Alveolar adenomas were identified in 20/40 cases (in 18/35 resections for primary lung cancer and two out of five resections for metastatic cancers).

*Incidence and degree of aneuploidy in tumours and preneoplastic lesions*

As demonstrated in Table II 31/35 (89%) of primary lung cancers (mean DI value 1.8, range 1.3–2.9) and three out of five (60%) of metastatic carcinomas were aneuploid. The incidences of aneuploidy in squamous cell carcinomas (93%) and adenocarcinomas (81%) were not judged to be statistically different ($P = 0.600$, Fisher's exact test). The incidences of aneuploidy in preneoplastic lesions associated with the central (57%) and peripheral (62%) tumours were also not judged significantly different ($P = 0.816$, chi-square test).

Aneuploidy in corresponding preneoplastic lesions was present in 18/31 (58%) of the aneuploid NSCLCs (Table III). Aneuploidy was absent in preneoplastic lesions associated with near-diploid tumours and with metastatic tumours. Thus only aneuploid tumours displayed aneuploidy in preneoplasia. An example of a typical DNA histogram is illustrated in Figure 3.

As also shown in Table III, aneuploid preneoplastic lesions were present throughout the central and peripheral compart-

**Table II** Frequency of aneuploidy in preneoplastic lesions

| Tumour type | *Number aneuploid/number tested (per cent)* No. aneuploid/ no. of tumours (%) | *Aneuploidy in one or more preneoplastic lesions* All tumours (%) | Diploid tumours | Aneuploid tumours (%) |
|---|---|---|---|---|
| Lung, all | 31/35 (89) | 18/35 (51) | 0/4 | 18/31 (58) |
| Squamous | 14/15 (93) | 8/15 (53) | 0/1 | 8/14 (57) |
| Adenocarcinoma | 13/16 (81) | 8/16 (50) | 0/3 | 8/13 (62) |
| Other | 4/4 (100) | 2/4 (50) | – | 2/4 (50) |
| Metastases | 3/5 (60) | 0/5 (0) | 0/2 | 0/3 (0) |

Forty tumours were examined, including 35 primary lung carcinomas and five pulmonary metastases from extrathoracic primary carcinomas.

**Table III** Location of aneuploid preneoplastic lesions

| Tumour location | *Location of aneuploidy in preneoplastic lesions* Any site (%) | Bronchi (%) | Bronchioles/alveoli (%) |
|---|---|---|---|
| NSCLC, all ($n = 31$) | 18 (58) | 5 (16) | 16 (52) (3/18 adenomas[a]) |
| Central ($n = 15$) | 9 (60) | 2 (13) | 8 (53) (1/9 adenomas) |
| Peripheral ($n = 16$) | 9 (56) | 3 (19) | 8 (50) (2/9 adenomas) |
| Metastases ($n = 3$) | 0 | 0 | 0 (0/2 adenomas) |

Because aneuploid preneoplastic lesions were not found in diploid tumours, the data presented are limited to the aneuploid tumour subsets. The aneuploid central NSCLCs included 14 squamous cell and one atypical carcinoid. The aneuploid peripheral NSCLCs included 13 adenocarcinomas, two large cell carcinomas and one adenosquamous carcinoma. [a]Aneuploidy was found in three alveolar adenomas, one associated with a central tumour and two with peripheral tumours.

**Figure 3** Representative DNA histogram demonstrating aneuploidy in a peripheral adenocarcinoma and in associated dysplastic alveolar type II cells. Several adenocarcinomas, including the one illustrated, demonstrated a broad, multipeak pattern. The associated dysplastic type II pneumocytes, several microscopic fields distant from the invasive carcinoma, have two aneuploid peaks. ▭, Lymphocytes; ▨, tumour cells; ▬, type II cells.

**Figure 4** Incidence and degree of aneuploidy in NSCLC and corresponding preneoplastic lesions. Dysplasia as previously indicated incorporates DI values for three aneuploid alveolar adenomas. (**a**) The percentage of lesions with aneuploidy. (**b**) Their respective mean DNA indices.

ments of the lung. However, the concentration of such lesions was greater in the periphery of the specimens, that is, in bronchioles and alveoli. The incidence and distribution for central and peripheral tumours were similar; the percentages for either compartment as well as for all tumours were approximately the same. Aneuploidy in bronchioles and pulmonary alveoli (peripheral compartment) was found in 53% of central tumours whereas in peripheral tumours, only 19% of the preneoplastic lesions were in bronchi (central compartment). From Tables I and III, it is apparent that the lesions of preneoplasia were concentrated in the peripheral compartment with no correlation with the anatomic site of origin of tumour.

*Incidence and degree of aneuploidy correlated with tissue lesion*

As demonstrated in Figure 4 the incidence and degree of aneuploidy increased in correlation with progressive severity of morphological change. In all aneuploid cases the DI values of the tumours were higher than those of their corresponding preneoplastic lesions. Aneuploidy was not found in histologically normal epithelium. A low level of aneuploidy was found in a minority of hyperplastic lesions, but the distinction between hyperplasia and mild dysplasia in Feulgen-stained microslides is difficult. Of 18 alveolar adenomas found in association with primary lung carcinomas, three (17%) were aneuploid (mean DI of 1.4). Two adenomas associated with metastatic cancers were diploid.

### Discussion

Understanding the molecular changes in the respiratory epithelium that precede the development of invasive cancers is crucial to the development of rational strategies for therapy and chemoprevention. Therefore, elucidation of the meaning of field cancerisation and its implications represents an important area in carcinogenesis research (Lippman *et al.*, 1993). In an effort to evaluate the relationship between preneoplastic changes and invasive carcinoma we determined the ploidy of the various preneoplastic changes found throughout the respiratory epithelium of patients with lung cancers.

Multifocal histopathological lesions are present in the respiratory epithelium of patients with lung cancer. A link has been established between smoking and radiation and the appearance and sequential progression of bronchial (central) preneoplastic lesions to invasive lung cancers (Auerbach *et al.*, 1957, 1962*b*; Saccomanno *et al.*, 1974). Histologically similar changes have been described even in the lungs of patients without cancer or in association with metastases to the lung from non-pulmonary primary cancers (Bernardi and Delsedime, 1989; Nakayama *et al.*, 1990; Weng *et al.*, 1992; Shimosato *et al.*, 1993). That moderate to severe squamous bronchial (central) dysplasia may spontaneously regress or never progress to invasive carcinoma has also been reported (Auerbach *et al.*, 1962*a*; Frost *et al.*, 1986).

Much less is understood about the relationship between the dysplastic changes of adenomatous hyperplasias in the peripheral parts of the lung and adenocarcinomas or other lung cancers. However, such lesions in peripheral lung are more common with adenocarcinomas (Weng *et al.*, 1990), suggesting they are indeed precancerous. Peripheral adenomas are alveolar lesions that are frequently found in association with adenocarcinomas and occasionally with other forms of NSCLC (Weng *et al.*, 1992). They have been postulated to be an intermediate step in the development of peripheral adenocarcinomas (Miller, 1990; Shimosato *et al.*, 1993). Since the malignant potential for many of these histopathological changes is not fully understood, for convenience, we refer to all of them as being preneoplastic.

We compared the frequency and degree of aneuploidy in preneoplastic lesions with their corresponding carcinomas. We found that 31 (89%) of 35 of the primary tumours were aneuploid with a mean DNA index (DI) of 1.8. This finding is in accord with previously published reports (Bunn *et al.*, 1983; Volm *et al.*, 1988). We observed aneuploidy in one or more preneoplastic lesions in 18 (51%) of 35 cases. However, aneuploidy was only present when the accompanying carcinoma was aneuploid (58% of the aneuploid tumour subset). Although some preneoplastic lesions had a DI greater than 1.5, in every instance, the DI of the tumour was greater than in the corresponding preneoplastic lesion. Three (60%) of five metastatic tumours were aneuploid, but none of the relatively mild preneoplastic lesions associated with metastatic tumours was aneuploid.

The degree and incidence of aneuploidy increased with progressive severity of histopathological change. Aneuploidy was occasionally observed in hyperplasia, usually appeared at the stage of dysplasia and then escalated along the postulated multistage pathway into invasive cancer. Although a low level of aneuploidy was detected in 20% of hyperplastic lesions, a distinction between hyperplasia and mild dysplasia in Feulgen-stained microslides is difficult. We believe that aneuploidy appears at the hyperplasia–dysplasia interface.

Our findings indicate that dysplastic lesions throughout the respiratory tree frequently demonstrate aneuploidy. Multiple lesions in bronchi (central compartment) and in bronchioles and alveoli (peripheral compartment) demonstrated aneuploidy. Of note, aneuploidy was found in central and peripheral lung compartments associated with both centrally and peripherally arising lung carcinomas. Thus the location of aneuploid lesions does not necessarily correlate with the presumed compartment of tumour origin. The presence of aneuploidy in peripheral foci is of interest because the natural history of these lesions is unknown. While aneuploidy in pulmonary preneoplasia has been reported by others (Nakayama *et al.*, 1990; Kogan *et al.*, 1991; Shimosato *et al.*, 1993), our report documents that it is extensive, especially in the peripheral parts of the lung. These findings provide support for the concept that the peripheral lesions are truly preneoplastic in nature.

Because aneuploidy can predispose to genomic instability (Loeb, 1991), it may be one of the predisposing causes of the multiple genetic abnormalities frequently present in invasive cancers. Reports of cytogenetic and other changes in preneoplastic bronchial lesions and even in 'normal' bronchial epithelium provide further evidence for this concept (Sozzi *et al.*, 1991, 1992). Recently, we have described deletions of the short arm of chromosomes 3 and 9 in hyperplastic lesions throughout the respiratory tree (Hung *et al.*, 1995). These chromosomal regions are the sites of known or putative recessive oncogenes important for the development of lung cancers of all histological types (Minna, 1993; Gazdar and Carbone, 1994). Thus aneuploidy may contribute to the multiple other genetic changes occurring in preneoplastic lesions long before the deveolopment of invasive cancer. Of interest, 3/20 (15%) of all alveolar adenomas examined were aneuploid. These data help validate the concept that many peripheral carcinomas may arise from alveolar adenomas, similar to the adenoma–carcinoma sequence present in colon cancer (Miller, 1990; Shimosato *et al.*, 1993).

Aneuploidy is present in multiple preneoplastic foci located throughout the respiratory epithelium from the stage of dysplasia onwards and increases in incidence and degree with progressive histopathological changes. These findings provide considerable support for the field cancerisation theory. Thus, aneuploidy may be a useful intermediate marker for assessing risk and monitoring the efficacy of chemoprevention trials.

**Acknowledgements**
We thank Donald McIntire, Academic Computing Services, University of Texas Southwestern Medical Center, for his assistance with statistical analyses.

### References

ANON. (1984). Early lung cancer detection: summary and conclusions. *Am. Rev. Respir. Dis.*, **130,** 565–570.

AUERBACH O, BREWSTER GJ, FORMAN JG, PETRICK TG, SMOLIN HJ, MUEHSAM GE, KASSOUNY DY AND STOUT AP. (1957). Changes in the bronchial epithelium in relation to smoking and cancer of the lung. *N. Engl. J. Med.*, **256,** 57–104.

AUERBACH O, HAMMOND EC AND GARFINKEL L. (1979). Changes in bronchial epithelium in relation to cigarette smoking, 1955–1960 *vs* 1970–1977. *N. Engl. J. Med.*, **300,** 381–385.

AUERBACH O, STOUT AP, HAMMOND EC AND GARFINKEL L. (1962*a*). Changes in bronchial epithelium in relation to smoking and cancer of the lung. *N. Engl. J. Med.*, **267,** 119–125.

AUERBACH O, STOUT AP, HAMMOND EC AND GARFINKEL L. (1962*b*). Bronchial epithelium in former smokers. *N. Engl. J. Med.*, **267,** 111–125.

BERNARDI P AND DELSEDIME D. (1989). Atypical changes of respiratory epithelium after heart-lung transplantation. *Pathol. Res. Pract.*, **184,** 514–518.

BOONE CW, KELLOFF GJ AND STEELE VE. (1992). Natural history of intraepithelial neoplasia in humans with implication for cancer chemoprevention strategy. *Cancer Res.*, **52,** 1651–1659.

BUNN PA, CARNEY DN, GAZDAR AF, WHANG-PENG J AND MATTHEWS MJ. (1983). Diagnostic and biological implications of flow cytometric DNA content analysis in lung cancer. *Cancer Res.*, **43,** 5026–5032.

CARSON FL. (1990). *Histotechnology.* ASCP Press: Chicago.

CARTER D. (1978). Pathology of the early squamous cell carcinoma of the lung. *Pathol. Annu.*, **13,** 131–147.

FEULGEN R AND VOIT K. (1924). Uber den Mechanismus der Nuclealfarbung. II Mittheilung. Uber das verhalten der kerne partiell hydrolysierter mikroskopischer Preparate zur fuchsinschwefligen Saure nach voraufgegangener Behandlung mit Phenylhydrazin. *Z. Physiol. Chem.*, **136,** 157–161.

FROST JK, BALL WJ, LEVIN ML, TOCKMAN MS, EROZAN YS, GUPTA PK, EGGLESTON JC, PRESSMAN NJ, DONITHAN MP AND KIMBALL AJ. (1986). Sputum cytopathology: use and potential in monitoring the workplace environment by screening for biological effects of exposure. *J. Occup. Med.*, **28,** 692–703.

GAZDAR AF AND CARBONE DP. (1994). *The Biology and Molecular Genetics of Lung Cancer.* R.G. Landes: Austin, TX.

GREENBERG SD. (1987). Carcinomas of the peripheral airways. In *Lung Carcinomas*, McDowell EM. (ed.) pp. 287–309. Churchill Livingstone: New York.

HEYNE KH, LIPPMAN SM, LEE JJ, LEE JS AND HONG WK. (1992). The incidence of second primary tumors in long-term survivors of small-cell lung cancer (see comments). *J. Clin. Oncol.*, **10,** 1519–1524.

HUNG J, KISHIMOTO Y, SUGIO K, VIRMANI A, MCINTIRE DD, MINNA JD AND GAZDAR AF. (1995). Allele-specific chromosome 3p deletions occur at an early stage in the pathogenesis of lung carcinoma. *JAMA*, **273,** 558–563.

JAGGI B, POON S, PONTIFEX B, FENGLER J, MARQUIS J AND PALCIC B. (1991). A quantitative microscope for image cytometry. *Spie*, **1448,** 89–97.

KOGAN EA, RODIONOV KV, TRISHKINA NV AND DUBROVSKAIA MI. (1991). [The DNA content in precancer, peripheral cancer and carcinoids of the lung (histospectrophotometric research)]. *Arkh. Patol.*, **53,** 17–24.

KUBIK A, PARKIN DM, KHLAT M, ERBAN J, POLAK J AND ADAMEC M. (1990). Lack of benefit from semi-annual screening for cancer of the lung: follow-up report of a randomized controlled trial on a population of high-risk males in Czechoslovakia. *Int. J. Cancer*, **45,** 26–33.

LIPPMAN SM, BENNER SE AND HONG WK. (1993). Chemoprevention strategies in lung carcinogenesis. *Chest*, **103,** 15S-19S.

LOEB LA. (1991). Mutator phenotype may be required for multistage carcinogenesis. *Cancer Res.*, **51,** 3075–3079.

MCCOMBE A, LUNG UJ AND HOWARD D. (1989). Multiple synchronous carcinomas of the aero-digestive tract. *J. Laryngol. Otol.*, **103,** 794–795.

MILLER RR. (1990). Bronchioloalveolar cell adenomas. *Am. J. Surg. Pathol.*, **14,** 904–912.

MILLER RR, NELEMS B, EVANS KG, MULLER NL AND OSTROW DN. (1988). Glandular neoplasia of the lung. A proposed analogy to colonic tumors. *Cancer*, **61,** 1009–1014.

MINNA J. (1993). The molecular biology of lung cancer pathogenesis. *Chest*, **103,** 449S–456S.

MINNA JD, GLATSTEIN EJ, PASS HI AND IHDE DC. (1989). Cancer of the lung. In *Cancer: Principles and Practice of Oncology*, DeVita VT Jr, Hellman S and Rosenberg SA. (eds.) pp. 591–705. Lippincott: Philadelphia.

NAKANISHI K. (1990). Alveolar epithelial hyperplasia and adenocarcinoma of the lung. *Arch. Pathol. Lab. Med.*, **114,** 363–368B.

NAKAYAMA H, NOGUCHI M, TSUCHIYA R, KODAMA T AND SHIMOSATO Y. (1990). Clonal growth of atypical adenomatous hyperplasia of the lung: cytofluorometric analysis of nuclear DNA content. *Mod. Pathol.*, **3,** 314–320.

NASIELL M AND KATO H. (1978). Cytomorphological grading and Feulgen DNA of metaplastic and neoplastic bronchial cells. *Cancer*, **41,** 1511–1521.

PALCIC B, JAGGI B AND MACAULAY C. (1990). The importance of image quality for computing texture features in biomedical specimens. *Spie*, **1205,** 155–162.

PARKIN DM, PISANI P AND FERLAY J. (1993). Estimates of the worldwide incidence of eighteen major cancers in 1985. *Int. J. Cancer*, **54,** 594–606.

SACCOMANNO G. (1982). Carcinoma *in situ* of the lung: Its development, detection and treatment. *Semin. Respir. Med.*, **4,** 156–160.

SACCOMANNO G, ARCHER VE, AUERBACH O, SAUNDERS RP AND BRENNAN LM. (1974). Development of carcinoma of the lung as reflected in exfoliated cells. *Cancer*, **33,** 256–270.

SAGMAN U, LISHNER M, MAKI E, SHEPHERD FA, HADDAD R, EVANS WK, DEBOER G, PAYNE D, PRINGLE JF, YEOH JL, GINSBERG R AND FELD R. (1992). Second primary malignancies following diagnosis of small cell lung cancer. *J. Clin. Oncol.*, **10,** 1525–1533.

SHIMOSATO Y AND MILLER RR. (1993). *Biopsy Interpretation of the Lung.* Raven Press: New York.

SHIMOSATO Y, NOGUCHI M AND MATSUMO Y. (1993). Adenocarcinoma of the lung: Its development and malignant progression. *Lung Cancer*, **9**, 99–108.

SLAUGHTER DP, SOUTHWICK HW AND SMEJKAL W. (1954). 'Field cancerization' in oral stratified squamous epithelium: Clinical implications of multicentric origin. *Cancer*, **6**, 963–968.

SOZZI G, MIOZZO M, TAGLIABUE E, CALDERONE C, LOMBARDI L, PILOTTI S, PASTORINO U, PIEROTTI MA AND DELLA-PORTA G. (1991). Cytogenetic abnormalities and overexpression of receptors for growth factors in normal bronchial epithelium and tumor samples of lung cancer patients. *Cancer Res.*, **51**, 400–404.

SOZZI G, MIOZZO M, DONGHI R, PILOTTI S, CARIANI CT, PASTORINO U, DELLA PG AND PIEROTTI MA. (1992). Deletions of 17p and p53 mutations in preneoplastic lesions of the lung. *Cancer Res.*, **52**, 6079–6082.

SUGIO K, KISHIMOTO Y, VIRMANI A, HUNG JY AND GAZDAR AF. (1994). K-*ras* mutations are a relatively late event in the pathogenesis of lung carcinomas. *Cancer Res.*, **54**, 5811–5815.

SUNDARESAN V, GANLY P, HASLETON P, RUDD R, SINHA G, BLEEHEN NM AND RABBITTS P. (1992). p53 and chromosome 3 abnormalities, characteristic of malignant lung tumours, are detectable in preinvasive lesions of the bronchus. *Oncogene*, **7**, 1989–1997.

VOLM M, BAK M, HAHN EW, MATTERN J AND WEBER E. (1988). DNA and S-phase distribution and incidence of metastasis in human primary lung carcinoma. *Cytometry*, **9**, 183–188.

WENG S, TSUCHIYA E, SATOH Y, KITAGAWA T, NAKAGAWA K AND SUGANO H. (1990). Multiple atypical adenomatous hyperplasia of type II pneumonocytes and bronchiolo-alveolar carcinoma. *Histopathology*, **16**, 101–103.

WENG SY, TSUCHIYA E, KASUGA T AND SUGANO H. (1992). Incidence of atypical bronchioloalveolar cell hyperplasia of the lung: relation to histological subtypes of lung cancer. *Virchows Arch. A Pathol. Anat. Histopathol.*, **420**, 463–471.